# Production of skimmed yogurt enriched by rice resistant starch: physicochemical properties, microscopic structure and model prediction during storage

**DOI:** 10.1016/j.fochx.2025.103205

**Published:** 2025-10-26

**Authors:** Weijie Qi, Meiyue You, Hongyue Zhao, Yaxing Xie, Bolatkhan K. Zayadan, Chiyu Yao, Jianjun Cheng, Qingfeng Ban

**Affiliations:** a College of Food Science, Northeast Agricultural University, Harbin 150030, China; b College of Data Science and Information Engineering, Harbin Huade University, Harbin 150025, China; c Faculty of Biology and Biotechnology, Al-Farabi Kazakh National University, Almaty 050040, Kazakhstan; d Heilongjiang Yihua Rice Industry Company Limited, Jiamusi 156300, China

**Keywords:** Low-glycemic yogurt, Resistant starch, Storage, Physicochemical properties, Microstructure

## Abstract

Resistant starch (RS) could be considered as a dietary fiber in yogurt, but its association with the shelf-life qualities is not clear. The characteristics and microstructure of low-glycemic yogurt (LGY) during fermentation and storage were investigated. During fermentation, principal component analysis combined with Mantel's test demonstrated that yogurt containing 0.6 % RS was best for storage. Over the 28-day storage, LGY maintained higher lactic acid bacteria count, with comparatively smaller reduction in microbial viability. LGY exhibited superior textural hardness (143.32 g) and elasticity (0.75). Microstructural analysis indicated that larger and looser pores appeared in both LGY and control over time, but the network cross-linking structure formed by RS with proteins resulted in less change in LGY. This structural reinforcement helped preserve the gel matrix and textural integrity. The kinetic model confirmed the storage stability of LGY. This provides a theoretical foundation for the stable LGY.

## Highlights

• Resistant starch effectively reduces the glycemic index of yogurt.
• Resistant starch enhances the structure and storage stability of yogurt.
• Kinetic models accurately predicts the shelf life of yogurt.

## Graphical abstract

**Figure:**
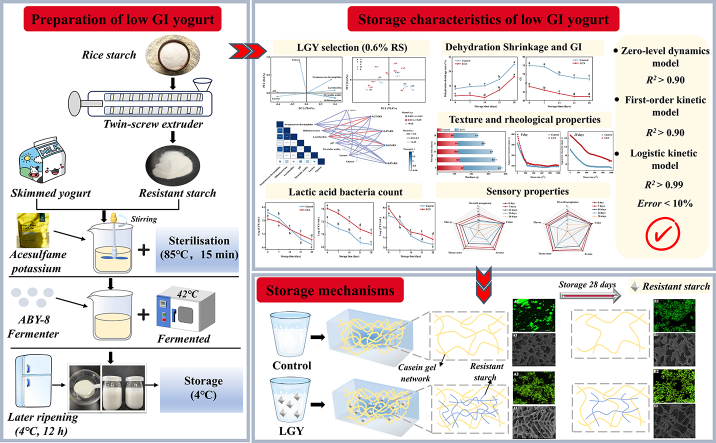

## 1 Introduction

Yogurt, a widely consumed fermented dairy product, is highly regarded for its richness in beneficial probiotics. The appearance, mouthfeel, viscosity and consistency of yogurt are the main texture attributes that influence consumers. However, yogurt products often encounter textural shortcomings such as low gel firmness and whey separation during storage. To mitigate these defects, strategies such as increasing total solids or protein content have been explored (Ares et al., 2007; Dircio-Morales et al., 2023; Pang et al., 2019). Additionally, polysaccharides are also widely employed. Wu et al. (2025) showed that kelp polysaccharides improved the adhesion and odor of solidified yogurt. Meanwhile, citrus pectin formed a complex with casein to produce a more stable protein gel structure, which improved its flavor profile and volatile compound composition (Yu et al., 2025). Among these approaches, the use of starch as a thickening agent has garnered significant interest due to its cost-effectiveness and its positive contribution to the structural integrity of yogurt. In yogurt matrices, starch typically retains partial granularity and interacts with milk proteins through crosslinking, thereby contributing to the formation of a structured acidified gel system. Nonetheless, conventional starches may promote the absorption of glucose at the site of the small intestine and the production of blood glucose, posing a potential health risk. This concern underscores the need for the incorporation of starches with reduced digestibility, which can promote a gradual glucose release and facilitate better glycemic control.

Rice starch is commonly used in dairy formulations as a fat replacer to partially replace milk fat, thereby reducing the overall fat content, while simultaneously contributing to the total solid content and helping to maintain the desired viscosity, mouthfeel, and creaminess. (Alting et al., 2009). Previous studies have demonstrated the successful preparation of rice resistant starch (RS) through physical, chemical, and enzymatic methods. RS exhibits functional characteristics akin to dietary fibers, including the ability to attenuate postprandial glycemic response, enhance satiety, and positively influence gut microbiota composition (He et al., 2019). Due to its capacity to retain a granular structure within emulsion gel systems, RS contributes to emulsion stability and can serve as a potential thickening agent, facilitating the development of yogurt products that are both nutritious and texturally appealing without compromising quality (Rezaei et al., 2015). Additionally, RS is recognized for its prebiotic properties, supporting the survival of probiotic cultures in yogurt under adverse storage or processing conditions (Etchepare et al., 2016). Currently, the application of prebiotics in yogurt is becoming increasingly widespread. Zhong et al. (2023) added lotus seed powder to milk and whey, significantly reducing lactose in dairy products. Costa et al. (2019) evaluated the addition of different polysaccharides as prebiotics to enhance yogurt flavor, but this approach diminished its quality. Notably, GI values have not been adequately evaluated. In the review by Zepeda-Hernández et al. (2021), prebiotics were mentioned to exert favorable hypoglycemic effects and enhance stability when incorporated into dairy products, yet further analysis is needed regarding the GI values of such products. Meanwhile, Szołtysik et al. (2021) confirmed that resistant starch stimulated the growth of probiotics in yogurt. Combining its excellent processing adaptability with minimal impact on food sensory quality, RS holds promise as an ideal ingredient for controlling yogurt texture and GI values.

In a study by Lee and Kang (2024), RS produced through heat-moisture treatment was applied to non-fat yogurt, resulting in enhanced viscosity, improved organoleptic qualities, and a significant reduction in whey separation. Similarly, Li et al. (2023) reported that the inclusion of high-amylose RS in lactogel formulations led to increases in viscosity, elastic modulus, and storage modulus, while the starch granules remained structurally intact within the gel matrix. These findings support the potential of RS as a functional ingredient in yogurt systems. Despite these promising results, current studies have not been comprehensive in evaluating the effects of RS on yogurt GI, lactic acid flora, texture, and sensory during storage. In particular, the influence of RS on the microstructural evolution of yogurt over time has yet to be elucidated. Moreover, the application of kinetic modeling to monitor changes in microbial populations and quality attributes offers a valuable method for predicting product stability throughout storage. Thus, the accurate determination of kinetic parameters is essential for understanding and managing the quality dynamics of yogurt containing RS during its shelf life.

Hence, the microstructural changes, GI stability, and kinetics of LGY during yogurt storage have not been thoroughly elucidated. The present study investigated the storage process of low-GI yogurt in detail through dynamic GI values, microstructural analysis, and a multi-indicator predictive model. In detail, the optimal RS addition concentration was selected from the fermentation results, and then the lactic acid bacteria counts, quality characteristics, rheological characteristics, and sensory characteristics of the low-GI yogurt were evaluated during the 28-day storage period. Meanwhile, the microstructural changes were observed by confocal laser scanning microscopy and cryo-transfer scanning electron microscopy to clarify the mechanism of quality retention. To accurately determine the shelf-life of yogurt, the kinetic study of quality parameter changes using zero and one-level kinetics as well as logistic prediction models was carried out. This could provide theoretical guidance for the development of highly stable and nutritious low-GI yogurts.

## 2 Materials and methods

### 2.1 Materials and low-GI yogurt preparation

Rice (*Oryza sativa*) was obtained from Wuchang City, Heilongjiang Province, China. The commercially available skim milk (fat content ≤0.5 % and protein content of 3.2 %) was provided by Mengniu Co., Ltd. (Harbin, China). The freeze-dried direct-to-vat starter culture ABY-8 (200 U) containing *Bifidobacterium B1–04*, *Lactobacillus bulgaricus ATCC 11842*, *Lactobacillus acidophilus LA-5*, and *Streptococcus thermophilus TH-4* was purchased from Chr. Hansen (Milwaukee, WI, USA). The *Lactobacillus plantarum LP28* (100 billion CFU/g) was purchased from Shenghe Biotechnology Co., Ltd. (Yangzhou, China). All other used reagents were analytical grade.

The broken rice was ground into rice flour and sieved through a 40-mesh screen for later use. A parallel corotating twin-screw extruder (Process 11, Thermo Scientific Co., USA) with an 11 mm diameter and an L/D ratio of 40:1 was employed for preparation. The moisture content was set at 24 %, screw speed at 130 rpm, feed rate at 5*g*/min, and extrusion temperature at 140 °C. After extrusion, the material was removed for starch extraction. The rice flour was wet-milled and passed through a 200-mesh sieve. 0.01 mol/L NaOH was added, followed by centrifugation. Protein was removed through multiple centrifugation steps. Ethanol was added, with stirring, followed by centrifugation to remove lipids and non-starch polysaccharides. The precipitate was dried at 40 °C for 12 h to prepare resistant starch (You et al., 2024). The total starch content was 862.21 mg/g, with resistant starch accounting for 33.79 %.

As shown in Fig. s1, 7 % (*w*/*v*) of acesulfame potassium was added to sterilized skimmed milk at 25 °C, along with different RS additions (0 %, 0.2 %, 0.4 %, 0.6 % and 0.8 %). The milk was then sterilized at 85 °C for 15 min. 0.03 % of ABY-8 ferment and 0.25 % of *Lactobacillus plantarum* powder were added at 25 °C. The milk was aseptically filled and fermented at 42 °C for 4 h. After completion of fermentation, it was left to post-ripen at 4 °C for 12 h to obtain the low-GI yogurt. Based on the changes in physicochemical properties during yogurt fermentation, a more ideal amount of resistant starch addition was selected. The low-GI yogurt was stored at 4 °C for 28 d for subsequent experiments (Paul et al., 2024). In storage, yogurt samples containing resistant starch were marked as LGY and yogurt without addition were marked as control.

### 2.2 Lactic acid bacteria and physicochemical characteristics of yogurt during fermentation

Plate counting method was used to investigate the changes of lactic acid bacteria population (Korkmaz et al., 2021), samples were taken at 0, 1, 2, 3 and 4 h of yogurt fermentation and the yogurt fermentation broth was diluted to 10^−6^, 10^−7^ and 10^−8^ as sample broths for preparation.

#### 2.2.1 Lactic acid bacteria count

(i) *Streptococcus thermophilus* counts.

The method was determined according to the method of Juan et al. (2024), with slight modification. The MC solid medium was employed. The composition of the modified MC solid medium included soybean peptone (5.0 g), beef extract powder (5.0 g), yeast extract powder (5.0 g), glucose (20.0 g), lactose (20.0 g), calcium carbonate (10.0 g), agar (15.0 g), and distilled water (1000 mL). The medium was adjusted to pH 6.1 using NaOH (0.1 mol/L) and sterilized at 121 °C for 20 min prior to use. Yogurt fermentation broth was serially diluted to 10^−6^, 10^−7^ and 10^−8^. 1 mL of the dilution was inoculated onto plates using the pour plate method, with three replicates for each dilution level. Additionally, 5 mL of 1 % neutral red solution was incorporated. The plates with colony count ranging from 30 to 300 CFU/plate were selected for enumeration. Meanwhile, the plates were then incubated in a constant-temperature incubator at 37 °C for 48 h. After calculating the average, the conversion is performed according to Eq. 1. Then the log(CFU/mL) is calculated.

(1) $$\mathrm{CFU}/\mathrm{mL}=\frac{\mathrm{Average colony count}\times \mathrm{Dilution factor}}{\mathrm{Inoculum volume}\;\left(\mathrm{mL}\right)}$$

(ii) Bifidobacterium counts.

It was determined according to the method of Vénica et al. (2025) with slight modification. The composition of the modified MRS solid medium was as follows: peptone (10.0 g), beef extract (10.0 g), dextrose (20.0 g), yeast extract (5.0 g), triammonium citrate (2.0 g), sodium acetate (5.0 g), dipotassium hydrogen phosphate (2.0 g), magnesium sulfate (0.1 g), manganese sulfate (0.05 g), Tween 80 (1.0 mL), and agar (15.0 g), all dissolved in 985 mL of distilled water. The pH was adjusted to 6.2, and mupirocin lithium salt (50 μg/mL) along with cysteine hydrochloride (500 μg/mL) were added to the solution, which was subsequently filtered and sterilized to a final volume of 1000 mL. The medium was then autoclaved at 121 °C for 15 min prior to use.

Yogurt fermentation broth was serially diluted to 10^−6^ and 10^−7^. 1 mL of the dilution was inoculated onto plates using the pour plate method, with three replicates for each dilution level. The plates were then incubated at 37 °C under anaerobic conditions for 72 h. The plates with colony count ranging from 30 to 300 CFU/plate were selected for enumeration. The conversion method is the same as Eq. 1.

(iii) Lactobacillus counts.

The preparation of the MRS medium followed the procedure described (ii), but it did not contain mupirocin lithium salt and cysteine hydrochloride. The remaining methods were the same as (ii).

#### 2.2.2 pH and titratable acidity

The yogurt was stirred into a runny liquid with a clean glass rod, and then the pH of the yogurt was measured by a pH meter (FE20, Mettler Toledo Instruments (Shanghai) Co., Ltd., China). For the determination of titratable acidity, 10.0 g of thoroughly mixed yogurt was accurately weighed and transferred into a clean conical flask. Subsequently, 20 mL of distilled water and 2.0 mL of phenolphthalein indicator solution were added. The mixture was then titrated with 0.1 mol/L NaOH until a persistent pale pink color was observed for at least 5 s (Saleh et al., 2020). The volume of NaOH standard solution consumed at this endpoint was recorded, and the titrimetric acidity was calculated using Eq. (2).

(2) $$\mathrm{Titratable acidity}\left({}^{{}^{\circ}}T\right)=\frac{10\times v_{\left(\mathrm{NaOH}\right)}\times 0.09\times 0.1}{m_{1}}\times 100$$

m_1_ is the weight of the sample, 0.1 is the molar concentration of NaOH, and 10 is the dilution of the sample, and 0.09 is the conversion factor for the acid.

#### 2.2.3 Lactose and glucose content

Lactose and glucose standards were accurately weighed and prepared into standard solutions at concentrations of 0.2, 2, 4, 6, 10, 12, 14, and 16 mg/g using ultrapure water. The solutions were subsequently filtered through a 0.45 μm aqueous filter membrane, and the filtrate was retained as the standard solution for further analysis. Yogurt samples (20 mg) were precisely weighed, followed by the addition of 4 mL of deionized water, 0.5 mL of zinc acetate solution, and 0.5 mL of potassium ferricyanide solution. The mixture was thoroughly homogenized and subjected to ultrasonic treatment for 30 min. The resulting supernatant was collected by centrifugation, filtered through a 0.45 μm aqueous filter membrane, and designated as the sample solution. Quantification of lactose and glucose was performed using a slightly modified version of the GB 5009.8–2023 method. An oscillometric refractive detector (Dionex UItiMate 3000, Thermo Scientific Co., Ltd., USA) was employed for the analysis. Separation was achieved using an amino acid chromatographic column (4.6 mm × 250 mm, 5 μm particle size) packed with aminosilane-bonded silica gel. Ultrapure water served as the mobile phase at a flow rate of 0.6 mL/min, with the column maintained at 60 °C and an injection volume of 10 μL (Gonzaga et al., 2019).

#### 2.2.4 GI value

10 g of yogurt with different amounts added resistant starch were dispersed in 10 mL of 0.9 % NaCl solution, and the pH was adjusted to 6. To simulate oral digestion, 0.3 mL of α-amylase (100 U/mg) was added, and the mixture was incubated at 37 °C with shaking for 2 min. After oral digestion, the pH was then adjusted to 2 using 0.1 mol/L HCl, followed by the addition of 0.3 mL pepsin (250 U/mg). The solution was shaken continuously at 100 rpm for 2 h at 37 °C. After gastric digestion, the pH was adjusted to 7 using NaOH, and then 0.8 mL trypsin (1000 U/mg) was added. The whole process was carried out at 37 °C with constant stirring. Enzymatic hydrolysates were collected at 0, 30, 60, 90, and 120 min. Meanwhile, the reaction was immediately terminated by adding 3 mL of 0.5 M Na_2_CO_3_ solution. Finally, centrifugation was performed at 5000 ×*g* for 10 min (Wang et al., 2023). The free glucose content was quantified using a GOPOD kit (BC2500). The GI values were calculated according to Eq. (3) (Mann et al., 2007). This method compared differences between samples based on in vitro simulated digestion and did not fully represent in vivo blood glucose changes.

(3) $$\mathrm{GI}=\frac{\mathrm{Value added area under the blood glucose curve for}\;2h\;\mathrm{of yogurt digestion}}{\mathrm{Value added area under the blood glucose curve}\;\mathrm{at}\;2h\;\mathrm{of equivalent glucose}}\times 100$$

### 2.3 Lactic acid bacteria and physicochemical properties of yogurt during storage

The enumeration of lactic acid bacteria during yogurt storage was conducted following the procedure outlined in Section 2.2.1. Measurements of pH and titratable acidity were performed in accordance with method 2.2.2, while GI values were determined as described in Section 2.2.4. Dehydration shrinkage was assessed by centrifuging the yogurt at 4 °C for 10 min at 2000 ×g. Subsequently, the supernatant was promptly removed and weighed (Amadarshanie et al., 2022). The degree of dehydration shrinkage was calculated using Eq. (4).

(4) $$\mathrm{Dehydration Shrinkage}\;\left(\%\right)=\frac{w_{\left(\mathrm{supernatant liquid}\right)}}{w_{\left(\mathrm{yogurt sample}\right)}}\times 100\%$$

### 2.4 Textural properties of yogurt during storage

The yogurt samples were removed from refrigeration at 4 °C and equilibrated to 25 °C prior to textural analysis. The yogurt was poured into cylindrical molds with a diameter of 15 mm and a height of 15 mm. The textural properties were assessed at 25 °C using an texture analyzer (SMS TA.XT plus, Stable. Micro Systems Co., Ltd., UK). Hardness, springiness, cohesiveness, gumminess and chewiness were measured employing an P36 probe. The test parameters were set at trigger force of 10 g, pretest speed of 6 mm/s, test speed of 6 mm/s, post-test speed of 6 mm/s, and distance traveled by probe of 10.00 mm (Wei et al., 2023).

### 2.5 Rheological properties of yogurt during storage

The rheological properties were determined according to the method of Wang et al. (2022) with minor modifications. Rheological analysis of the samples was conducted using a Rheonaut rheometer (HAAKE MARS 40, Thermo Scientific Co., Ltd., USA). A parallel plate measurement system with a diameter of 35 mm and a gap spacing of 0.1 mm was employed for the tests. The apparent viscosity of the yogurt samples was evaluated as a function of shear rate over a range of 0.1 to 100 s^−1^. Frequency sweep tests were performed within a frequency range of 0.1 to 10 Hz at a controlled temperature of 25 °C, with a strain value set at 1 %. Additionally, strain sweep tests (0.01–100 %) were conducted to determine the linear viscoelastic region of the samples.

### 2.6 Sensory evaluation of yogurt during storage

Sensory evaluation of the yogurt products was conducted by ten trained assessors (five men and five women) from the laboratory who had successfully completed professional basic training. The evaluation encompassed five attributes: color, aroma, tissue state, flavor and overall acceptability. To minimize bias, the two groups of yogurt samples were randomly coded. The assessment was performed under standardized conditions, with an ambient temperature of 25 °C, adequate lighting, and the absence of extraneous odors (Sun et al., 2020). The detailed scoring criteria are presented in Tab s1. In addition, prior to conducting the sensory evaluation, these group members were informed of the purpose of the experiment and the composition of the samples (all edible and posing no food safety risks). They were also made fully aware of and agreed to be subjects of the sensory analysis study. For more detailed information, please refer to the ethical statement.

### 2.7 Laser scanning confocal microscopy of yogurt during storage

The microstructural characteristics of yogurt during storage were examined using laser confocal electron microscopy (CLSM) (FV3000, Olympus Co., Ltd., Japan). A 0.1 g sample of yogurt was placed onto a glass slide for analysis. Protein, starch, and fat components were selectively stained using Nile blue (0.5 %, *w*/*v*), fluorescein isothiocyanate (FITC, 0.7 %, w/v), and Nile red (0.2 %, w/v), respectively, with isopropanol serving as the solvent. Subsequently, 10 μL of each staining solution was added to the sample, and the reaction was conducted in the dark for 5 h. The excitation/emission wavelengths for Nile blue, FITC, and Nile red were set at 633/655–695 nm (green), 488/490–530 nm (yellow), and 488/530–590 nm (red), respectively (Devnani et al., 2022).

### 2.8 Cryo-scanning electron microscope of yogurt during storage

The microstructural analysis of yogurt was conducted following the methodology described by Ban et al. (2020). The yogurt samples were rapidly frozen using liquid nitrogen. The frozen samples were then transferred to a preparation chamber, where they were fractured using a cold scalpel. Subsequently, the fractured samples underwent an etching process at −85 °C for 20 min, followed by gold sputter-coating. The microstructural characteristics of yogurt were then observed using cryo-scanning electron microscopy (SU8010, Hitachi Co., Ltd., Japan) at an accelerating voltage of 5 kv.

### 2.9 Storage model predictions of yogurt

The changes in quality indicators during storage showed distinct trends over time, reflecting the quality trends at each moment during storage. This was considered a dynamic response to storage duration. Zero-order and first-order kinetics applied to storage processes where a single variable change caused property alterations. The logistic model was used for microbial non-exponential decay processes, better capturing inflection points changes. Three models enabled comprehensive comparison and analysis of yogurt quality changes.

#### 2.9.1 Zero-order and first-order kinetics models

The employment of zero-order and first-order kinetics for predicting changes in the physicochemical properties of yogurt with storage time (Sun et al., 2022). The specific formulas were Eq. (5) and Eq. (6):

(5) $$\mathrm{Zero}-\mathrm{order}:f\left(x\right)=f\left(x_{0}\right)-\mathrm{kx}$$

(6) $$\mathrm{First}-\mathrm{order}:f\left(x\right)=f\left(x_{0}\right)\exp\left(-\mathrm{kx}\right)$$

Where f(x) was the value of the quality indicator, x was the storage time, f(x_0_) was the initial quality index value of the yogurt, f(x) was the quality index value of the yogurt at x time, and k was the reaction rate constant.

#### 2.9.2 Logistic model

Meanwhile, for the sake of scientific validity of the test and accuracy of the prediction, the Logistic model could also be used to predict the change of yogurt in storage well (Nor-Khaizura et al., 2019). The Logistic model was specified as Eq. (7).

(7) $$f\left(x\right)=f\left(x_{0}\right)+\left(f_{\left(\max\right)}-f\left(x_{0}\right)\right)/\left(1+{\left(x/x_{0}\right)}^{p}\right)$$

Where f(x) was the value of the quality indicator, f(x_0_) was the initial quality index value, f_(max)_ was the final quality index, x was the storage time (d), x_0_ was the time (d) corresponding to the inflection point of the quality index value, and *p* was the rate constant for the change of the quality index value.

### 2.10 Statistical analysis

Analysis of variance (ANOVA) was employed to compare differences between treatments. The measurements were performed multiple times for each sample. Tukey's HSD test was conducted using SPPS 16.0 software. The significance of the differences was established at *P <* *0.05*. The Pearson correlation analysis, principal component analysis (PCA), and kinetic analysis were performed using origin 2021 (Origin Lab Corporation, USA). Mantel test was performed using Chiplot.

## 3 Results and discussion

### 3.1 Lactic acid bacteria of yogurt during fermentation

#### 3.1.1 *Streptococcus thermophilus*

**Fig. 1:**
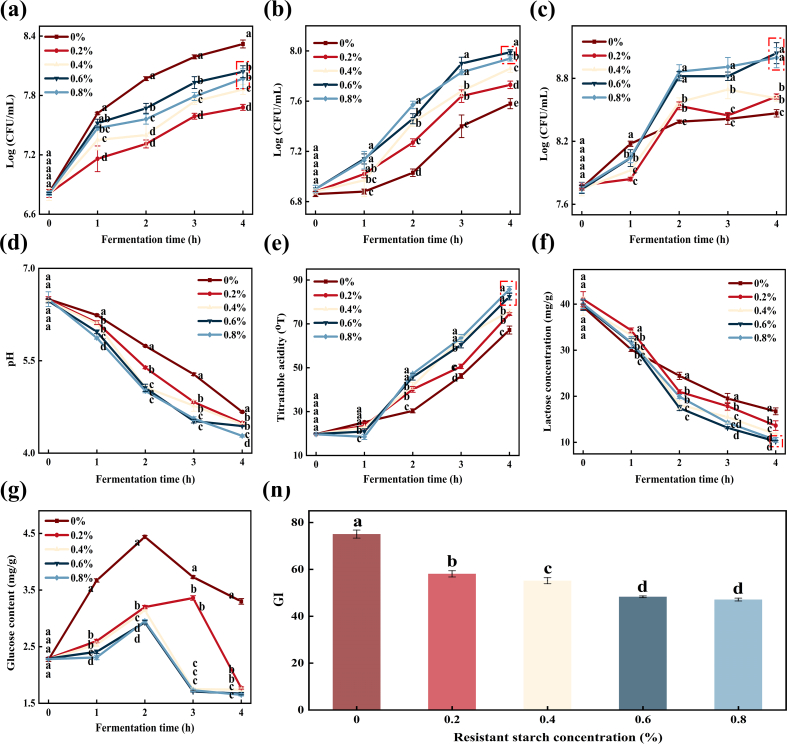
The lactic acid bacteria and physicochemical properties of yogurt with different resistant starch additions during the fermentation. (a) *Streptococcus thermophilus* count, (b) *Bifidobacterium* count, (c) *Lactobacillus* count, (d) pH, (e) titratable acidity, (f) lactose content, (g) glucose content, (h) GI of yogurt fermented for 4 h. Note: The different lowercase letters (a-e) indicate comparisons between the same fermentation time points.

The influence of varying additions of RS on the growth of *Streptococcus thermophilus* during yogurt fermentation is illustrated in Fig. 1a. Across all stages of fermentation, the counts of *Streptococcus thermophilus* in yogurts containing RS were consistently lower than those in the control. This could be due to *Streptococcus thermophilus* relying on lactose for growth, with the presence of RS indirectly affecting lactose availability, while other probiotics may have competed for nutrients within the system. Specifically, during the initial 0–1 h of fermentation, all samples exhibited a rapid increase, indicating an initial growth advantage. However, after the first hour, the growth rate of *Streptococcus thermophilus* in RS-containing yogurts notably slowed. This phenomenon may be attributed to the interaction between *Streptococcus thermophilus* and other lactic acid bacteria, wherein *Streptococcus thermophilus* plays a dominant role in acid production during the early stages of fermentation. The resulting pH reduction facilitates the release of polypeptides and free amino acids by other lactic acid bacteria, resources that are progressively depleted and utilized as fermentation proceeds, consequently leading to a deceleration in *Streptococcus thermophilus* proliferation (Arioli et al., 2017). Among the RS treatments, the addition of 0.6 % RS resulted in the highest increase in *Streptococcus thermophilus* counts (8.06 %). By the end of fermentation, the differences in *Streptococcus thermophilus* counts between yogurts containing 0.6 % and 0.8 % RS were minimal. Therefore, an RS addition level of 0.6 % appears to be optimal for supporting the growth of *Streptococcus thermophilus* while incorporating RS into yogurt formulations.

#### 3.1.2 Bifidobacterium

Fig. 1b illustrates the effect of varying additions of RS on the growth of *Bifidobacterium* during yogurt fermentation. Throughout the fermentation process, yogurts supplemented with RS consistently exhibited higher counts compared to the control, indicating that RS promotes the proliferation of *Bifidobacterium*. This could be contributed to by *Bifidobacteria* possessing a more diverse array of carbohydrate-active enzymes, enabling them to efficiently degrade and utilize RS as both a carbon source and energy source, thereby promoting their own growth and reproduction. In the initial stage of fermentation, no significant differences (*P* > 0.05) bacterial counts were observed among the samples. However, as fermentation progressed, the growth rate of *Bifidobacterium* in RS-enriched yogurt surpassed that of the control. More specifically, during the 0–1 h period, the growth of *Bifidobacterium* was limited, as they were undergoing an adaptation phase. Following this period, a marked increase in *Bifidobacterium* counts was observed in RS-containing yogurts, which may be attributed to the availability of oligosaccharides generated by RS, facilitating the proliferation. After 3 h of fermentation, the growth of *Bifidobacterium* plateaued. This stabilization may be due to the accumulation of organic acids resulting from bacterial metabolism, which can create an unfavorable acidic environment, thereby inhibiting further bacterial multiplication and reducing sugar utilization (Peng et al., 2022). Notably, yogurt supplemented with 0.6 % RS achieved a *Bifidobacterium* count of 7.97 log (CFU/mL) at 4 h, higher than both the control and other RS-treated groups. This finding suggests that 0.6 % RS addition provides an optimal condition for *Bifidobacterium* growth during yogurt fermentation.

#### 3.1.3 Lactobacillus

Fig. 1c illustrates the impact of varying additions of RS on *Lactobacillus* growth during yogurt fermentation. The yogurt containing RS showed higher overall growth rates than the Control during fermentation. This was due to Lactobacillus effectively utilizing RS as a high-quality carbon source, resulting in faster growth rates. Compared to the control, RS-supplemented yogurts exhibited a slower initial growth of *Lactobacillus* during the 1 h. However, between 1 and 2 h, the growth rate accelerated markedly, surpassing that of the control. After 2 h, the growth rate plateaued, with bacterial counts reaching their maximum at 4 h. Notably, the final *Lactobacillus* counts in RS-enriched samples were higher than those in the control. This enhanced growth may be attributed to the acidification of the yogurt matrix caused by the metabolic activity of *Lactobacillus*, which facilitates its own proliferation. The effect is likely due to the enzymatic breakdown of RS during fermentation, yielding oligosaccharides that serve as fermentable substrates for *Lactobacillus*. Meanwhile, the rough surface morphology of RS granules may provide favorable sites for bacterial adhesion, thereby facilitating their proliferation (Salem et al., 2013). Additionally, after 1 h of fermentation, *Lactobacillus* growth accelerated as the RS concentration increased. This may be attributed to RS producing acid during fermentation, lowering the pH of system. The acid hydrolysis of proteins increased free amino acids and peptide substances, thereby promoting microbial growth (Keser & Ozcan, 2025). Although the most substantial increase in *Lactobacillus* counts occurred in yogurts containing 0.6 % and 0.8 % RS, the difference between these two groups was minimal (*P* > 0.05). This may be explained by the accumulation of metabolic by-products, such as formic acid and carbon dioxide, in the later fermentation stages, which can reduce pH and subsequently inhibit *Lactobacillus* proliferation.

### 3.2 Physicochemical properties of yogurt during fermentation

#### 3.2.1 pH and titratable acidity

The pH variation during the fermentation of yogurts supplemented with different additions of RS was presented in Fig. 1d. Across all samples, a general downward trend in pH was observed over the fermentation period. During the initial stage, the pH values among the different treatments exhibited minimal variation. However, from 1 h onward, a noticeable divergence emerged between the control and RS-enriched yogurt (*P* < 0.05). Specifically, the control exhibited the greatest overall pH reduction, declining from 6.50 to 4.67 over the 4 h period. By the end of fermentation (4 h), the pH values of RS-containing yogurts had stabilized and declined to below 4.5. These findings suggest that the incorporation of RS enhances the rate of acid production during fermentation. This accelerated acidification likely facilitates faster protein coagulation and gel formation, thereby contributing to improved structural integrity and texture development in RS-fortified yogurts compared to the control.

As illustrated in Fig. 1e, the titratable acidity of all yogurt samples exhibited an increasing trend throughout the fermentation. After 1 h of fermentation, although significant differences (*P* < 0.05) were observed, the numerical variations were not substantial. However, after 1 h, the titratable acidity increased markedly across all treatments, with variations in acid production rates observed depending on the concentration of RS added. The higher RS levels were associated with a more rapid increase in titratable acidity. By the end of the 4 h fermentation period, the RS-fortified yogurts exhibited titratable acidity values exceeding 70.00°T, whereas the control demonstrated a comparatively lower value of 67.19°T. This indicates that RS supplementation accelerates acid development and promotes the early formation of the yogurt gel matrix. Among the RS-supplemented yogurt, those containing 0.6 % and 0.8 % RS achieved the highest acid production. However, the difference between these two groups was not statistically significant (*P* > 0.05). Hence, to reduce costs, the 0.6 % RS addition achieves the same effect as higher concentrations.

#### 3.2.2 Lactose content and glucose content

The variation in lactose content of yogurts with different additions of RS during fermentation is presented in Fig. 1f. At the onset of fermentation, the lactose content across all yogurt samples was approximately 40.07 mg/g. As fermentation progressed, a general declining trend in lactose content was observed in all treatments. Notably, during the initial 0–1 h of fermentation, the rate of lactose depletion in RS-supplemented yogurts was lower than the control. This phenomenon may be attributed to the presence of RS prolonging the adaptation phase of lactic acid bacteria. After 1.5 h of fermentation, the lactose content in the control yogurt declined at a slower rate, whereas the lactose reduction rate in RS-supplemented yogurts accelerated. This accelerated lactose degradation is likely associated with the increased acidity and elevated counts of lactobacilli, as RS addition promotes the proliferation of lactobacilli, thereby enhancing lactose metabolism (Sharma et al., 2023). Among all samples, yogurt supplemented with 0.6 % RS exhibited the most rapid decrease in lactose content, achieving a reduction of 74.32 %. By the end of the 4-h fermentation period, the lactose concentrations in yogurts containing 0.6 % and 0.8 % RS had decreased to the lowest levels, reaching approximately 10.00 mg/g.

During yogurt fermentation, lactose is hydrolyzed by lactic acid bacteria into glucose and galactose. The changes in glucose content in yogurts with varying additions of RS during fermentation are illustrated in Fig. 1g. In the control, the glucose content exhibited an initial increase followed by a decline over the course of fermentation. In this case, except for the yogurt with 0.2 % RS, the glucose content of all samples increased during 0–2 h of fermentation. This trend may be attributed to incomplete utilization of glucose following lactose hydrolysis by lactic acid bacteria. By 3 h of fermentation, the glucose content in yogurts containing more than 0.4 % RS began to decrease. After 4 h of fermentation, the glucose content in RS-supplemented yogurts was lower than that in the control group, with a progressive decrease in glucose accumulation corresponding to increasing RS concentrations. This phenomenon can be explained by the stimulatory effect of RS on lactic acid bacteria proliferation, which enhances glucose consumption and utilization.

#### 3.2.3 GI values

The GI values of yogurts with different additions of RS at the end of fermentation are presented in Fig. 1h. After 4 h of fermentation, significant differences (*P* < 0.05) were observed in the GI values among the yogurt samples with varying RS concentrations. The GI value of the control yogurt was 75.07, categorizing it as a high-GI food. In contrast, yogurts supplemented with 0.2 % and 0.4 % RS exhibited GI values of 58.12 and 55.20, respectively, classifying them as medium-GI foods. This reduction in GI is attributed to the RS-induced proliferation of lactic acid bacteria, which enhances sugar metabolism and accelerates lactose degradation (Jia et al., 2022). Moreover, yogurts containing 0.6 % and 0.8 % RS demonstrated GI values of 48.34 and 47.14, respectively, both falling within the low-GI category, with no significant difference (*P* > 0.05) observed between these two groups. This outcome is primarily due to the higher RS content promoting a greater abundance of lactic acid bacteria, thereby facilitating increased consumption and utilization of lactose and glucose, ultimately leading to reduced glucose accumulation in the yogurt system. The in vitro model for predicting the glycemic response of carbohydrate foods is considered a potentially useful approach. However, while the use of in vitro digestion was justified in Wang et al. study (2023) when compared to in vivo glucose changes, further clinical trials are still needed to validate these findings.

### 3.3 PCA analysis and mantel test of yogurt during fermentation

#### 3.3.1 PCA analysis

**Fig. 2:**
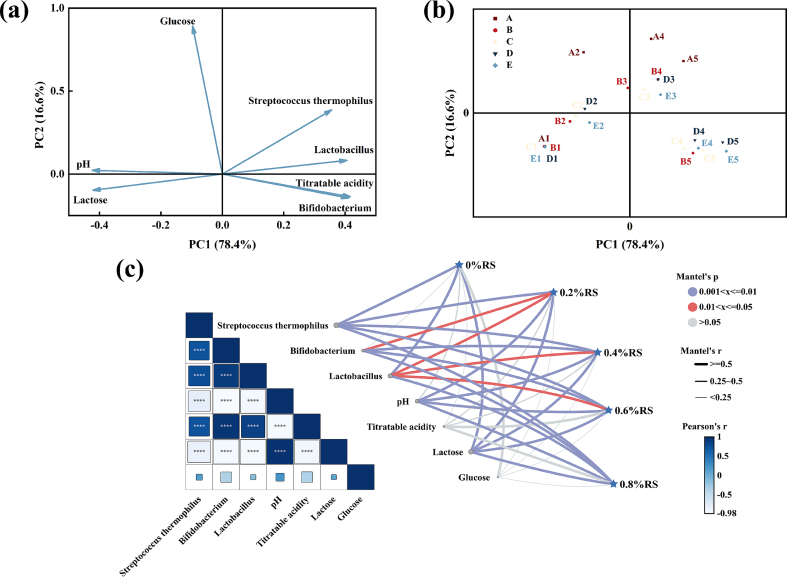
Principal component analysis and Mantel test for the quality of yogurt with different resistant starch additions during the fermentation process. (a) load chart, (b) score chart, (c) correlation analysis and mantel test. Note: In Fig. b, A, B, C, D and E represent resistant starch additions of 0 %, 0.2 %, 0.4 %, 0.6 % and 0.8 %, respectively. *X1*, *X2*, *X3*, *X4*, *X5* represent fermentation for 0 h, 1 h, 2 h, 3 h and 4 h, respectively. For example, A1 represents yogurt with 0 % RS addition fermented for 0 h. In Fig. c, X%RS represents combinations at X% RS addition with different fermentation times.

PCA was conducted to evaluate the physicochemical and microbial metrics of yogurts supplemented with varying additions of RS during fermentation. As illustrated in Fig. 2a and b, the first two principal components (PC1 and PC2) accounted for 78.4 % and 16.6 % of the cumulative variance, respectively. PC1 encompassed the majority of the variation associated with yogurt quality attributes, whereas PC2 primarily captured variations related to *Streptococcus thermophilus* counts and glucose content. At the initial stage of fermentation, no statistically significant differences (*P* > 0.05) were observed among samples in PC1. However, as fermentation progressed, distinct separations among the samples became evident in the PCA plot. Importantly, at 4 h of fermentation, yogurts supplemented with 0.6 % and 0.8 % RS exhibited no differences along either PC1 or PC2, yet they were clearly differentiated from the control group.

#### 3.3.2 Mantel test

To further validate the optimal level of RS addition, correlation analysis and Mantel tests were conducted based on the combination of RS addition levels, fermentation time, and yogurt quality indicators (Fig. 2c). The color gradient in the correlation matrix represents pairwise correlations between quality indicators, where dark blue squares indicate strong positive correlations and light blue squares represent negative correlations. Additionally, the color intensity and width of the connecting lines between RS additions and quality indicators illustrate the statistical significance and the Mantel test r-statistic (Sunagawa et al., 2015). The correlation analysis revealed that pH was negatively correlated with lactic acid bacteria counts, supporting the hypothesis that lactic acid bacteria proliferation reduces pH. Moreover, a positive correlation was observed among the different *Lactobacillus* strains, indicating a synergistic interaction during fermentation. Lactose content exhibited a negative correlation with all indicators except pH, suggesting that after 4 h of fermentation, lactose was almost completely utilized as the lactic acid bacteria population reached its peak. The Mantel test demonstrated a strong overall correlation between RS addition-fermentation time combinations and yogurt quality indicators (Mantel's *r* > 0.5, *P* < 0.01). Compared to the control group, the association between RS addition and both lactic acid bacteria counts and lactose content strengthened with higher RS additions. Although lactose showed a slightly enhanced correlation (r > 0.5) at 0.6 % and 0.8 % RS, the results were not statistically significant (*P* > 0.05). In contrast, glucose content did not correlate with RS addition or fermentation time. Overall, yogurts supplemented with 0.6 % and 0.8 % RS demonstrated significant correlations with lactobacilli counts, pH, and lactose content (r > 0.5, *P* < 0.05), as well as strong correlations with titratable acidity (r > 0.5), indicating that these formulations maintained superior quality during fermentation.

Based on PCA and Mantel test results, no significant difference (*P* > 0.05) was observed between yogurt with 0.6 % RS and 0.8 % RS additions. Notably, the 0.6 % RS yogurt exhibited slightly higher lactic acid bacteria counts after 4 h fermentation compared to the 0.8 % RS yogurt, while achieving equivalent GI values. To reduce raw material costs in actual processing, the 0.6 % RS addition rate could serve as a preferable option.

### 3.4 Lactic acid bacteria of LGY during storage

#### 3.4.1 *Streptococcus thermophilus*

**Fig. 3:**
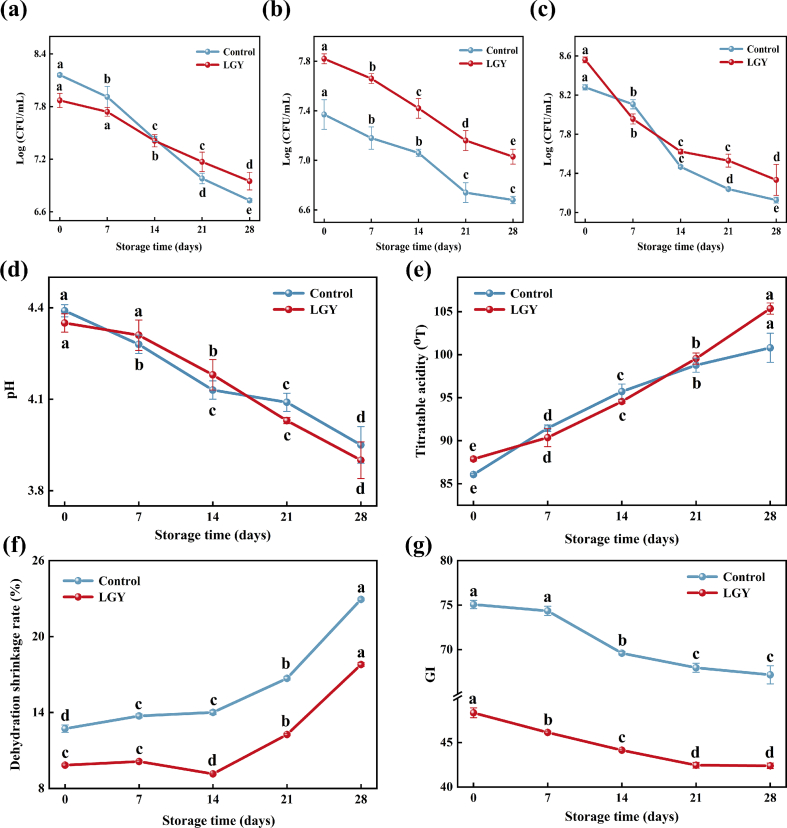
The changes in lactic acid bacteria and physicochemical properties of yogurt during storage. (a) *Streptococcus thermophilus* count, (b) *Bifidobacterium* count, (c) *Lactobacillus* count, (d) pH, (e) titratable acidity, (f) dehydration shrinkage rate, (g) GI. Note: Different lowercase letters (a-e) indicate significant differences (*P*<0.05) in the mean values within the same parameter group.

The changes in *Streptococcus thermophilus* counts in LGY during 28 d of storage are presented in Fig. 3a. Under the same counting unit, no significant differences (*P* > 0.05) were observed in the *Streptococcus thermophilus* counts of LGY within the first 7 d, whereas a significant decline (*P* < 0.05) was detected in the control over the same period. From 0 d to 14 d, the *Streptococcus thermophilus* counts in the control yogurt remained higher than those in LGY. Notably, after 14 d of storage, the counts in LGY surpassed those of the control. This phenomenon can be attributed to the increased acidity and depletion of available nutrients in the control yogurt during the later stages of storage, both of which adversely affected the growth and viability of *Streptococcus thermophilus*.

#### 3.4.2 Bifidobacterium

The changes in *Bifidobacterium* counts of LGY during 28 d of storage are illustrated in Fig. 3b. At the beginning of storage, the *Bifidobacterium* counts in LGY and the control were 7.82 log (CFU/mL) and 7.39 log (CFU/mL), respectively. Throughout the 28-day storage period, both samples exhibited a declining trend in *Bifidobacterium* counts. This decrease may be attributed to the initial abundant utilization of nutrients such as oligosaccharides by *Bifidobacterium*, followed by a gradual depletion of these substrates over time, ultimately leading to a reduction in *Bifidobacterium* viability. Notably, *Bifidobacterium* counts remained consistently higher in LGY compared to the control throughout storage. It is hypothesized that the presence of RS contributed to this phenomenon by releasing additional small-molecule carbon sources, such as maltose and glucose, under acidic conditions, thereby sustaining higher basal metabolic activity in bifidobacteria and mitigating the decline associated with carbon source depletion (Rengadu et al., 2021).

#### 3.4.3 Lactobacillus

The changes in *Lactobacillus* counts of LGY during 28 d of storage are presented in Fig. 3c. Under the same counting unit, both LGY and the control exhibited a significant decline in *Lactobacillus* counts over the storage period (*P* < 0.05). LGY showed a rapid decrease in *Lactobacillus* counts within the first 7 d, likely due to the depletion of nitrogen sources necessary for bacterial growth during storage. After 7 d, *Lactobacillus* counts in both groups continued to decrease. However, the rate of decline in LGY was slower compared to the control. The availability of small-molecule carbon sources released from RS likely helped maintain relatively higher metabolic activity in LGY. After 14 d of storage, the *Lactobacillus* count in LGY was slightly higher than that of the control yogurt, which demonstrated the beneficial effects of RS.

### 3.5 Physicochemical properties of LGY during storage

#### 3.5.1 pH and titratable acidity

The changes in pH of LGY during the storage are shown in Fig. 3d. Both the control and LGY exhibited a decreasing trend in pH over the 28-day storage period. In the control yogurt, the pH declined significantly (*P* < 0.05) from an initial value of 4.39 to 3.95. In contrast, the pH of LGY remained relatively stable during the first 7 d, with no significant difference observed (*P* > 0.05). However, by day 28, it had decreased significantly (*P* < 0.05) to 3.91. Notably, after 14 d of storage, the rate of pH decline in LGY surpassed that of the control. This phenomenon may be attributed to the gradual release of fermentable carbon sources from the resistant starch, either through relaxation of its physical structure or enzymatic degradation, thereby stimulating the metabolic activity of lactic acid bacteria and enhancing the conversion of lactose into lactic acid (Li et al., 2024).

The changes in titratable acidity of LGY during storage are presented in Fig. 3e. Both the control and LGY exhibited an overall increasing trend in titratable acidity throughout the storage period, with values remaining within an acceptable range (70–110°T). During the 7–14 d of storage, the titratable acidity of the control was higher than LGY. However, after 14 d, the titratable acidity of LGY surpassed that of the control. This observation suggests that the addition of RS promotes a greater increase in yogurt acidity during the later stages of storage, likely due to the sustained growth and metabolic activity of lactic acid bacteria in the LGY, leading to continued acid production.

#### 3.5.2 Dehydration shrinkage rate

Dehydration shrinkage refers to the contraction of the gel matrix in solidified yogurt, resulting in the separation of whey. The changes in dehydration shrinkage of LGY during storage are presented in Fig. 3f. For both the control and LGY, dehydration shrinkage increased progressively with extended storage time. The initial dehydration shrinkage of the control yogurt was 12.72 %, and a significant increase (*P* < 0.05) was observed throughout the storage period, reaching a maximum of 22.94 % by 28 d. This trend indicates structural instability of the control yogurt, leading to gel contraction and subsequent whey separation. In contrast, the initial dehydration shrinkage of LGY was 9.84 %, with a significant increase (*P* < 0.05) to a maximum of 17.79 % at day 28. Notably, LGY consistently exhibited lower dehydration shrinkage compared to the control during storage. This improvement is primarily attributed to the addition of resistant starch, which functions as a thickening agent, enhancing the protein and total solids content of the yogurt and thereby mitigating dehydration shrinkage (You et al., 2024).

#### 3.5.3 GI values

The variation in the GI values of yogurt during the storage period is presented in Fig. 3g. As shown in the figure, all yogurt samples exhibited a decreasing trend in GI values over time. Specifically, the GI value of the control yogurt declined from 75.07 to 67.17, transitioning from the high GI range to the medium GI range. A significant difference (*P* < 0.05) in GI values was observed at 14 d of storage. This suggests that the lactic acid bacteria present in the control yogurt partially contributed to a continuous decrease in GI by metabolizing lactose and other substrates. The extent of this decrease was relatively limited. In the case of LGY, the initial GI value at day 0 was 48.34, which already categorized it as a low-GI food. A significant reduction (*P* < 0.05) in GI was detected at day 7, and the GI value further decreased to 42.45 by day 21, with no significant difference (*P* > 0.05) observed between days 21 and 28. This trend can be attributed to the ongoing metabolism of sugars by lactic acid bacteria during storage. Nevertheless, in the later stages of storage, the increasingly acidic environment inhibited the growth and survival of lactobacilli, and the depletion of available nutrients further limited their activity, ultimately resulting in a stabilization of the GI values.

### 3.6 Textural properties of LGY during storage

**Fig. 4:**
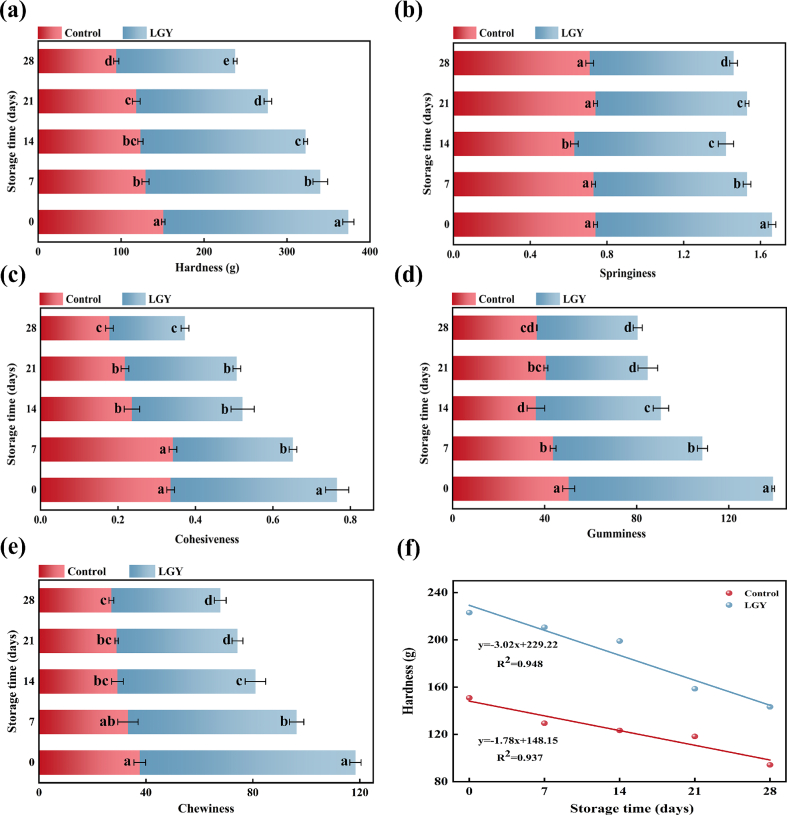
The textural characterization of yogurt during storage. (a) hardness, (b) springiness, (c) cohesiveness, (d) gumminess, (e) chewiness, (f) linear fitting of hardness. Note: Different lowercase letters (a-d) indicate significant differences (*P*<0.05) in the mean values within the same parameter group.

The changes in hardness, springiness, cohesiveness, gumminess, and chewiness of LGY during 28 d of storage are shown in Fig. 4. Regarding hardness, both the control and LGY exhibited a decreasing trend over time. The hardness values of LGY exceeded 200 g up to 14 d of storage and remained consistently higher than those of the control. Notably, the rate constant for hardness reduction in the control yogurt (1.78) was lower than that of LGY yogurt (3.02). Conversely, the extent of reduction in LGY (35.87 %) was smaller than control (37.33 %). This phenomenon may be attributed to the formation of a uniformly distributed starch-protein cross-linked structure within LGY, which enhanced the overall hardness of the yogurt matrix.

Throughout the storage period, no significant difference (*P* > 0.05) in the springiness of the control yogurt was observed between 0 and 7 d. However, springiness decreased to 0.63 at 14 d and subsequently increased to approximately 0.73 by days 21 and 28. Compared to LGY, the springiness values of the control fluctuated more markedly, likely due to the poorer structural stability of the system. Conversely, LGY exhibited a more stable and modest decline in springiness, with values ranging from 0.92 to 0.75. This enhanced stability is likely due to the increased total solids content induced by RS addition, which promoted a more crosslinked protein micelle network within the yogurt matrix. The cohesiveness of LGY also showed a decreasing trend, with significant differences (*P* < 0.05) observed during storage. In the control yogurt, cohesiveness remained stable between days 0 and 7 but exhibited significant changes thereafter (*P* < 0.05). Gumminess properties, which reflect the energy required to break the yogurt matrix into swallowable fragments, were consistently lower in the control yogurt compared to LGY, with both groups showing significant (*P < 0.05*) changes over time. Furthermore, although the chewiness of LGY decreased during the 28-day storage period, it consistently remained higher than that of the control yogurt. These results may be attributed to the fact that RS is linked to the surrounding proteins to form a homogeneous and stable network structure, which increases the binding capacity of casein macromolecules and improves the gelation capacity of the yogurt system, thus showing strong cohesiveness, gumminess, and chewiness (Sun et al., 2019).

### 3.7 Rheological properties of LGY during storage

**Fig. 5:**
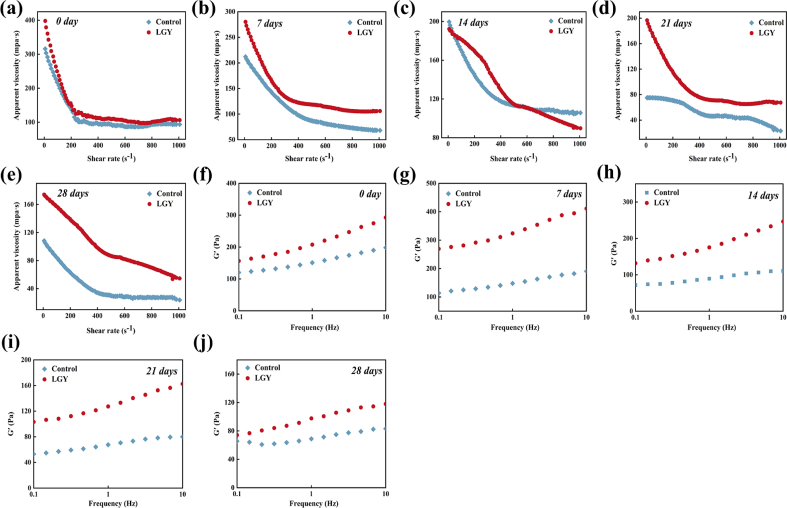
The rheological property of yogurt during storage. a-e represent the apparent viscosity of yogurt stored for 0, 7, 14, 21 and 28 d, respectively. f-j represent the elastic modulus of yogurt stored for 0, 7, 14, 21 and 28 d, respectively.

The changes in apparent viscosity during the storage of LGY are presented in Fig. 5a–e. Both yogurt groups exhibited a decreasing trend in apparent viscosity with increasing shear rate, demonstrating typical shear-thinning behavior. This phenomenon can be attributed to the disentanglement of molecular chains and the disruption of aggregates within the yogurt matrix under shear stress. During the 28-day storage period, both LGY and the control exhibited a decreasing trend in apparent viscosity, with LGY consistently maintaining a higher viscosity than the control yogurt. Initially, the apparent viscosity of LGY was 398.02 mPa·s, whereas that of the control yogurt was 315.91 mPa·s. As storage time progressed, both yogurt groups experienced a reduction in apparent viscosity, though to varying extents. On day 7, LGY maintained a higher apparent viscosity than the control, primarily due to the increased total solids content introduced by RS addition and the associated enhancement in water-holding capacity. By day 14, the apparent viscosity of LGY had decreased to 189.25 mPa·s, approaching the value observed in the control group. Notably, at day 21, the apparent viscosity of LGY exhibited a slight increase and remained higher than that of the control yogurt. This increase was likely due to the ability of RS to bind casein micelles more effectively, thereby reinforcing the gel structure and improving the viscoelastic properties of the yogurt system. By day 28, although the apparent viscosity of LGY declined again, it remained greater than that of the control yogurt. Overall, these findings indicate that the incorporation of RS enhanced the structural stability and rheological properties of the yogurt compared to the control.

The modulus of elasticity (G′) of the frequency scans accurately expresses the elasticity of yogurt. The elasticity changes of LGY are shown in Fig. 5f-j. The G′ value of LGY was higher than that of control yogurt throughout the storage period, indicating that RS as an added ingredient could improve the elasticity of yogurt. This is mainly due to the fact that swollen starch granules act as fillers in the protein network, thus enhancing the cross-linking properties of the protein network (Sun et al., 2019). In particular, the G′ of LGY showed a fluctuating trend during 28 d of storage, but was always higher than that of the control. The G′ was elevated at 7 d of storage and showed a continuous decreasing trend from 14 to 28 d of storage. On the contrary, G′ of the control group decreased continuously from day 0. Thus, the appropriate addition of RS resulted in increased interaction forces in the yogurt system and facilitated the strengthening of the gel structure of yogurt compared with control yogurt.

### 3.8 Sensory evaluation of LGY during storage

**Fig. 6:**
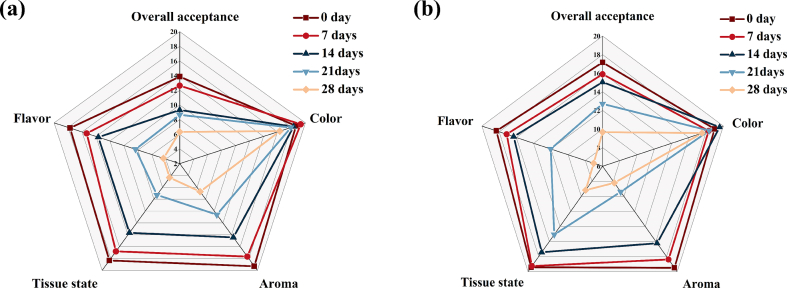
The sensory evaluation of yogurt during storage. (a) control, (b) LGY.

Sensory evaluation is one of the important indexes for assessing the quality of yogurt. The results of sensory scores of the control group and LGY are displayed in Fig. 6. With the extension of storage time, all the sensory indexes of yogurt in both groups showed a decreasing trend. For the overall scores, at 0 d of storage, the scores of both yogurt groups were the highest values. After that, the overall scores decreased with the extension of storage time, but the scores of LGY were higher than those of the control. This indicates that the addition of RS is beneficial to improve the quality of yogurt. Meanwhile, the scores in flavor of LGY were always higher than the control during storage. As the storage time increased, the flavor scores showed a decreasing trend. This was due to the higher acidity of the control yogurt and the poor growth of lactic acid bacteria. The tissue state of the control yogurt showed a rapid decreasing trend throughout the storage period, whereas the tissue state of LGY showed a smaller decreasing trend. This is mainly due to the increase in acidity of the yogurt during the storage period, the cross-linking of protein particles in the system to form larger aggregates, resulting in increased structural porosity, and the cross-linking of proteins by the RS to form a gel network structure, which increases the consistency of the yogurt, which is in turn beneficial to the tissue state of the yogurt. During the storage period, the aroma of the yogurts in both groups showed a decreasing trend. During the 14 d of storage, the control yogurt was close to the LGY score, and then the odor score of the control yogurt decreased faster than that of the LGY. This was due to the increase in the acidity value of the yogurt system, which resulted in a pronounced sour odor. The color scores of both groups of yogurt remained high during the storage period, which indicates that there was no significant change (*P* > 0.05) in the color of the yogurt, both of which showed a creamy state.

### 3.9 CLSM microstructure of LGY during storage

**Fig. 7:**
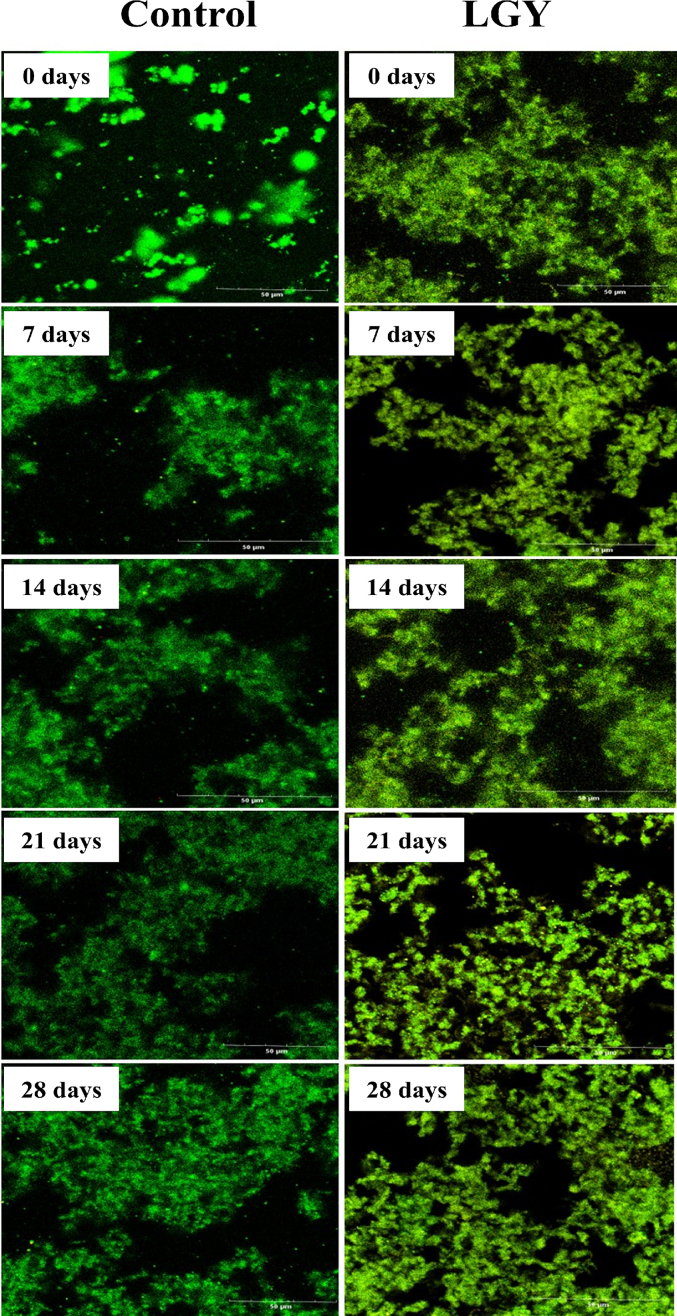
The confocal laser scanning electron microscopy of yogurt during storage. Note: Proteins of yogurt are colored green and resistant starch is colored yellow. (For interpretation of the references to color in this figure legend, the reader is referred to the web version of this article.)

The microstructural changes in LGY, as observed through CLSM, are depicted in Fig. 7. At the onset of the storage period, the control yogurt exhibited a structure characterized by dispersed granules that were widely spaced apart. In contrast, LGY displayed uniformly distributed starch–protein cross-linked structures, with a more compact cross-linking network compared to the control. This suggests that the incorporation of RS improved the pore size and uniformity of the cross-linked structure within the yogurt matrix. This enhancement may be attributed to the increased solid content in LGY due to the addition of RS, as well as the even distribution of swollen starch granules that became cross-linked with the protein network during fermentation. Between days 7 and 14 of storage, no notable changes were observed in the microstructure of LGY. However, between days 21 and 28, the protein cross-linking structure of the control yogurt became thinner, with larger voids observed in the matrix. Although the tissue structure of LGY also exhibited an increase in pore size during this period, starch granules remained present within the protein voids, contributing to the consolidation of the yogurt structure. These findings suggest that the RS–protein cross-linking network plays a crucial role in stabilizing the yogurt gel system throughout the storage period.

### 3.10 Cryo-SEM of LGY during storage

**Fig. 8:**
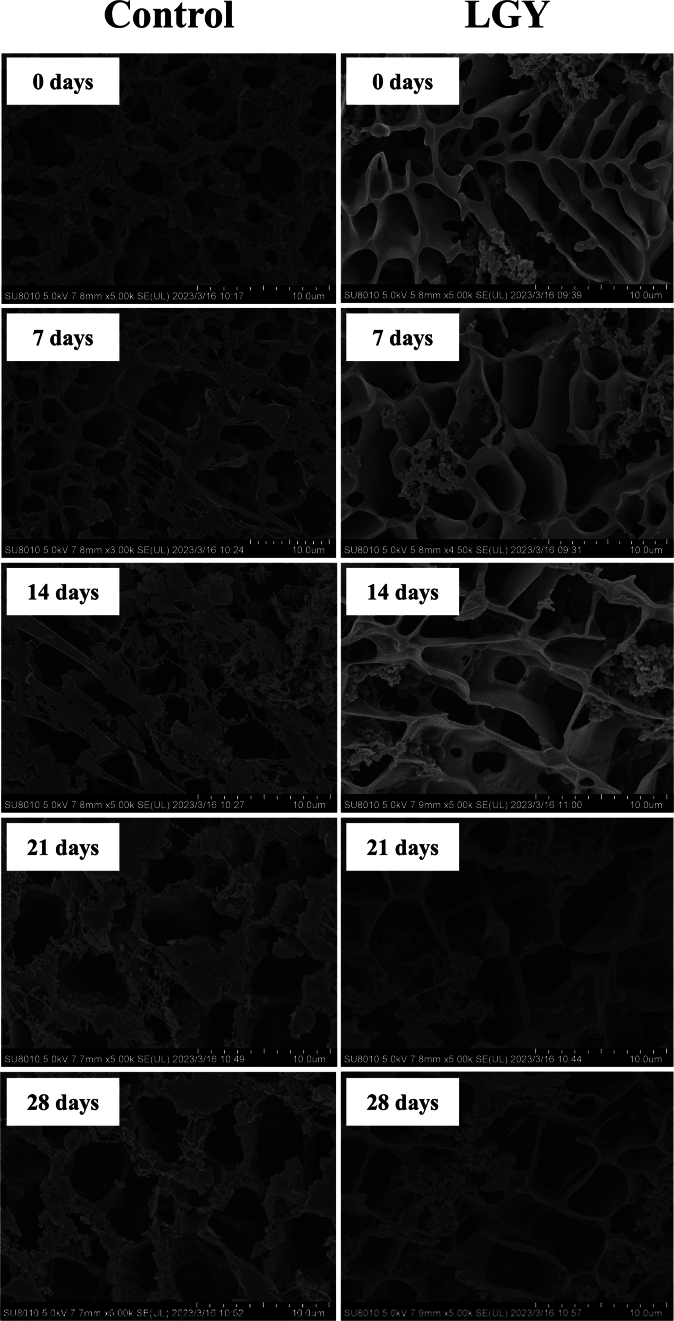
The cryo-scanning electron microscope of yogurt during storage.

The cryo-SEM images of LGY are presented in Fig. 8. As depicted, both yogurt groups exhibit clear protein micelles and open pores within their structures. Over the course of storage, the pore size in both yogurt systems gradually increased. The elongated pores were observed in the control yogurt. In comparison, LGY displayed a denser pore structure and a more extensively cross-linked gel network. Additionally, starch agglomerates were embedded within the protein voids, suggesting that proteins interact with RS through hydrophobic and hydrogen bonding interactions, thereby forming a homogeneous network structure within the yogurt matrix. After 7 d of storage, the control yogurt showed signs of strain between the micellar structures, indicating that the network cross-linking was weak, rendering it more susceptible to deformation and rupture. In contrast, which indicates that there was no significant (*P* > 0.05) change in the color of the yogurt, both of which showed a creamy state changes were observed in the micellar cross-linking structure of LGY. At 14 d, small, loose voids appeared in the protein network of the control yogurt, and these voids progressively enlarged. While similar enlargements were observed in the protein voids of LGY, some of these voids were filled with amylose-protein granule agglomerates, which helped to maintain the integrity of the protein cross-linking structure. By day 28, the structure of the control yogurt had undergone severe degradation, whereas the network structure of LGY exhibited larger pores. This may be attributed to a decrease in the pH of the yogurt system, which promoted increased aggregation between proteins, resulting in larger gaps within the matrix. In conclusion, while the gel structure of yogurt deteriorated over time, leading to the formation of larger pores, the addition of RS contributed to the preservation of a stable gel structure and enhanced the overall quality of the yogurt during storage.

### 3.11 Storage prediction model analysis

#### 3.11.1 Kinetics in the quality of yogurt

The kinetic order of storage was determined by fitting the experimental data for various quality indicators to different kinetic models, and the results are presented in Tab. s2 and 3. The suitability of each model was assessed using the coefficient of determination (r^2^), with higher r^2^ values indicating a stronger correlation between the experimental data and the model predictions. The quality indicators, including *Streptococcus thermophilus*, *Bifidobacterium*, *Lactobacillus*, pH, titratable acidity, GI, and hardness. All exhibited zero and first-order kinetic behavior, with r^2^ values exceeding 0.9. However, the fitting coefficient for dehydration shrinkage rate was relatively low. For *Lactobacillus*, the k-values for LGY were consistently higher than those for the control, suggesting that the addition of RS enhanced the growth and proliferation of probiotics. Additionally, the observed acidity levels support the notion that the small molecular carbon sources provided by RS created a favorable environment for the growth of Lactobacillus and facilitated acid production. The GI value of LGY exhibited a slower rate of change compared to the control, while the change in hardness for LGY was more pronounced. This can be attributed to the initially looser structure of the control yogurt, which displayed a smoother change. Despite the more rapid changes observed in LGY, the actual hardness values remained consistently higher for LGY than for the control yogurt. The k-values derived from secondary kinetic models showed a similar trend to those obtained from primary kinetics.

#### 3.11.2 Logistic model in the quality of yogurt

The kinetic behavior of *Streptococcus thermophilus*, *Bifidobacterium*, *Lactobacillus*, titratable acidity, and GI of yogurt was modeled using a logistic model. In contrast to the zero and first-order kinetics, pH and hardness did not conform to this model. As shown in Tab. s4, the coefficient of determination (R^2^) values was all greater than 0.99, and the relative errors were less than 10 %, indicating a good fit of the model to the data. The *p*-values for *Streptococcus thermophilus*, *Bifidobacterium*, and *Lactobacillus* in LGY were higher than those in the control group, suggesting that the addition of RS provided a small molecular carbon source that helped maintain the activity of lactic acid bacteria during storage. The rate of increase in titratable acidity was higher for LGY compared to the control, which can be attributed to the gradual release of fermentable carbon sources from RS due to structural relaxation or enzymatic degradation. This process likely stimulated the metabolic activity of lactic acid bacteria and accelerated the acidification rate. Regarding GI values, the rate of decrease for LGY during storage was much slower than that of the control group, although the GI of the control group remained in the high GI range.

In conclusion, all three models provided excellent fits for the changes in probiotic counts within the yogurt system. Compared to the others, the Logistic model demonstrated superior fitting ability, accurately capturing the inflection points in probiotic dynamics. However, it failed to adequately account for the physicochemical properties of yogurt. The zero-order and first-order kinetic models exhibited the opposite behavior. No differences were observed between these two approaches.

## 4 Conclusions

The incorporation of RS into yogurt has garnered considerable interest due to its potential to produce low GI products. The study validated the feasibility of RS supplementation in yogurt to enhance its storage stability and glycemic regulatory potential, thereby supporting its commercial application in addressing the nutritional requirements of individuals with hyperglycemia. During fermentation, PCA and Mantel tests indicated that a 0.6 % RS addition promoted the proliferation of lactic acid bacteria, reduced GI values, and improved physicochemical properties. Throughout storage, RS-enriched yogurt maintained higher probiotic viability, exhibited reduced whey separation, and consistently remained within the low-GI classification. Compared with the control, the texture and elasticity of LGY were better preserved. Sensory evaluation further revealed greater overall acceptability for LGY, suggesting that RS contributed positively to consumer perception of its texture and flavor. CLSM and Cryo-SEM indicated that all yogurts developed larger pores during storage, yet RS promoted the strengthening of protein interstitials, resulting in a more stable structure of LGY. Kinetic modeling (zero-order, first-order, and logistic models) accurately predicted the shelf life of yogurt, highlighting the enhanced storage stability imparted by RS. Acting as a functional thickening agent, RS improved the overall quality and shelf stability of yogurt, and the findings of this study offer theoretical support for the development and storage, low-GI dairy products. Meanwhile, the fine structure and in vivo digestion of LGY during storage warrant further investigation to validate its low GI efficacy.

## CRediT authorship contribution statement

**Weijie Qi:** Writing – review & editing, Writing – original draft, Methodology, Data curation, Conceptualization. **Meiyue You:** Investigation, Formal analysis, Data curation. **Hongyue Zhao:** Formal analysis, Data curation. **Yaxing Xie:** Conceptualization. **Bolatkhan K. Zayadan:** Formal analysis, Data curation. **Chiyu Yao:** Supervision. **Jianjun Cheng:** Writing – review & editing, Supervision, Methodology. **Qingfeng Ban:** Writing – review & editing, Data curation, Conceptualization.

## Ethical statement

The sensory evaluation in this study has been carried out in accordance with the ethical and professional guidelines as outlined in the IFST (the Institute of Food Science & Technology, UK) Guidelines for Ethical and Professional Practices for the Sensory Analysis of Foods. Informed consent was obtained from all participants involved in the study. The authors declare that throughout the study, we made every effort to protect the rights and privacy of all participants. This included ensuring that participants were not coerced into testing, providing information about the study requirements and associated risks, obtaining written consent from participants, avoiding the disclosure of participant data without their knowledge, and allowing participants to withdraw from the study at any time. Although there was no formal ethics committee, the study consistently adhered to the principles and guidelines set forth by the IFST to ensure ethical treatment of participants.

## Declaration of competing interest

The authors declare that they have no known competing financial interests or personal relationships that could have appeared to influence the work reported in this paper.

## Data Availability

Data will be made available on request.

## Appendix A Supplementary data

Supplementary material Image 1
