# Supplementary material for: Production of skimmed yogurt enriched by rice resistant starch: physicochemical properties, microscopic structure and model prediction during storage
Source: Food Chem X. 2025 Oct 26;31:103205. doi: 10.1016/j.fochx.2025.103205 (PMC12613054; doi:10.1016/j.fochx.2025.103205)
Supplement: Supplementary file 1 — Supplementary material [file mmc1.docx]

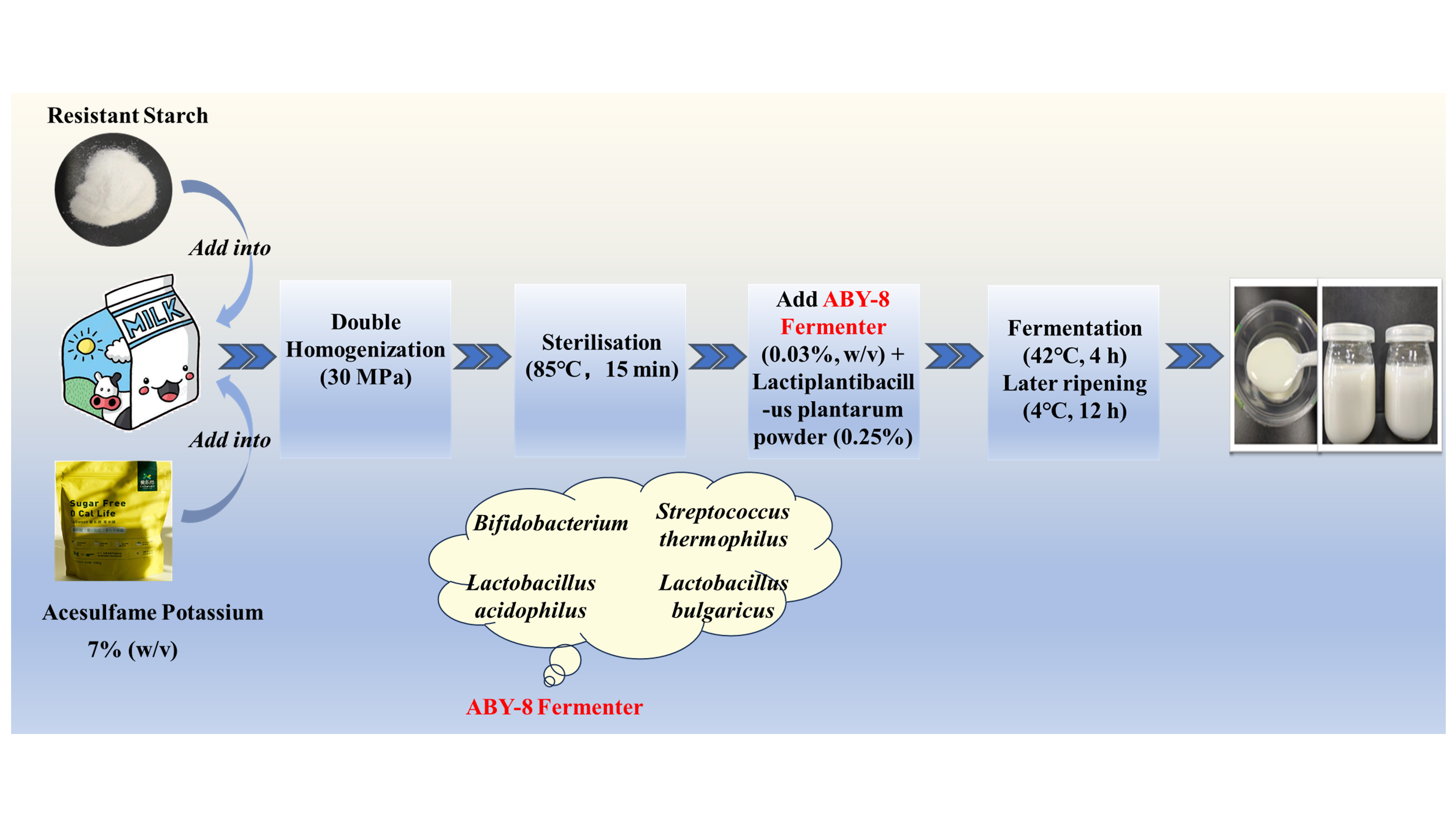


**Figure s1.** The flow chart for the preparation of low GI yogurt.

**Table s1.** Sensory evaluation scoring table.

| Indicators | Evaluation standards | Scores |
| --- | --- | --- |
| Tissue state (20) | Homogeneous curd, good consistency, no sedimentation and layering | 10~20 |
|  | Homogeneous curd, good consistency, slight sedimentation and layering | 5~10 |
|  | Uneven curd, too thick or too thin, sediment stratification is obvious | ＜5 |
| Aroma (20) | Clear fermented yogurt flavor, no bitterness, no off-flavors | 10~20 |
|  | Normal aroma, slightly bitter, no off-flavors | 5~10 |
|  | Heavier bitterness, off-flavors | ＜5 |
| Flavor (20) | Moderately sweet and sour, no bitterness | 10~20 |
|  | Sour or slightly sweet, slightly bitter taste | 5~10 |
|  | Excessive sourness or sweetness, bitter taste | ＜5 |
| Color (20) | Colors creamy white, no miscellaneous colors | 10~20 |
|  | Slightly yellow in color | 5~10 |
|  | Dark, yellowish or slightly brownish in color | ＜5 |
| Overall acceptability (20) | Delicate and smooth taste, sweet and sour, viscous, no roughness | 10~20 |
|  | More delicate and slightly rough on the palate | 5~10 |
|  | Lack of finesse, too thin, roughness apparent | ＜5 |

**Table s2.** Zero-order parameters of the changes of yogurt stored under different times.

| Quality indicators | Samples | k | R^2^ |
| --- | --- | --- | --- |
| ***Zero-order kinetic model*** | |  |  |
| *Streptococcus thermophilus* | Control | 0.034 | 0.987 |
|  | LGY | 0.057 | 0.983 |
| *Bifidobacterium* | Control | 0.026 | 0.966 |
|  | LGY | 0.030 | 0.989 |
| *Lactobacillus* | Control | 0.041 | 0.931 |
|  | LGY | 0.046 | 0.897 |
| pH | Control | 0.015 | 0.979 |
|  | LGY | 0.017 | 0.971 |
| Titratable acidity | Control | -0.526 | 0.968 |
|  | LGY | -0.631 | 0.980 |
| Dehydration shrinkage rate | Control | -0.334 | 0.799 |
|  | LGY | -0.257 | 0.653 |
| GI | Control | 0.317 | 0.920 |
|  | LGY | 0.223 | 0.934 |
| Hardness | Control | 1.783 | 0.948 |
|  | LGY | 3.021 | 0.937 |

**Table s3.** First-order kinetic parameters of the changes of yogurt stored under different times.

| Quality indicators | Samples | k | R^2^ |
| --- | --- | --- | --- |
| ***First -order kinetic model*** | |  |  |
| *Streptococcus thermophilus* | Control | 0.005 | 0.986 |
|  | LGY | 0.007 | 0.986 |
| *Bifidobacterium* | Control | 0.004 | 0.967 |
|  | LGY | 0.004 | 0.988 |
| *Lactobacillus* | Control | 0.005 | 0.937 |
|  | LGY | 0.006 | 0.909 |
| pH | Control | 0.004 | 0.980 |
|  | LGY | 0.004 | 0.966 |
| Titratable acidity | Control | -0.006 | 0.959 |
|  | LGY | -0.007 | 0.987 |
| Dehydration shrinkage rate | Control | -0.023 | 0.863 |
|  | LGY | -0.025 | 0.793 |
| GI | Control | 0.004 | 0.923 |
|  | LGY | 0.005 | 0.943 |
| Hardness | Control | 0.014 | 0.923 |
|  | LGY | 0.016 | 0.927 |

**Table s4.** Logistic parameters of the changes of yogurt stored under different times.

| Quality indicators | Samples | Fitted equation | R^2^ | Relative error (%) |
| --- | --- | --- | --- | --- |
| ***Logistic models*** | |  |  |  |
| *Streptococcus* *thermophilus* | Control | f(x)=6.17+1.98/(1+(x/17.88)^2.12^) | 0.999 | 0.96 |
|  | LGY | f(x)=6.48+1.40/(1+(x/20.29)^2.03^) | 0.998 | 1.35 |
| *Bifidobacterium* | Control | f(x)=6.03+1.33/(1+(x/25.79)^1.91^) | 0.970 | 5.21 |
|  | LGY | f(x)=6.63+1.19/(1+(x/19.46)^1.57^) | 0.998 | 0.75 |
| *Lactobacillus* | Control | f(x)=7.09+1.19/(1+(x/11.33)^3.55^) | 0.999 | 9.64 |
|  | LGY | f(x)=6.54+2.02/(1+(x/17.82)^0.88^) | 0.995 | 5.28 |
| Titratable acidity | Control | f(x)=110.30-24.24/(1+(x/19.51)^1.22^) | 0.999 | 0.21 |
|  | LGY | f(x)=2272.26-2184.40/(1+(x/872.52)^1.40^) | 0.999 | 1.14 |
| GI | Control | f(x)=67.13+7.98/(1+(x/11.71)^4.22^) | 0.998 | 4.61 |
|  | LGY | f(x)=41.21+7.11/(1+(x/10.88)^1.92^) | 0.992 | 9.46 |

Note: pH, dehydration shrinkage rate and hardness did not fit the logistic model. Relative error was (measured value - true value) / true value.
